# Scopoletin: a review of its pharmacology, pharmacokinetics, and toxicity

**DOI:** 10.3389/fphar.2024.1268464

**Published:** 2024-02-23

**Authors:** Xiao-Yan Gao, Xu-Yang Li, Cong-Ying Zhang, Chun-Ying Bai

**Affiliations:** ^1^ Basic Medicine College, Chifeng University, Chifeng, China; ^2^ Inner Mongolia Key Laboratory of Human Genetic Disease Research, Chifeng University, Chifeng, China; ^3^ Key Laboratory of Mechanism and Evaluation of Chinese and Mongolian Pharmacy at Chifeng University, Chifeng University, Chifeng, China

**Keywords:** scopoletin, plant sources, pharmacological activities, pharmacokinetics, toxicology

## Abstract

Scopoletin is a coumarin synthesized by diverse medicinal and edible plants, which plays a vital role as a therapeutic and chemopreventive agent in the treatment of a variety of diseases. In this review, an overview of the pharmacology, pharmacokinetics, and toxicity of scopoletin is provided. In addition, the prospects and outlook for future studies are appraised. Scopoletin is indicated to have antimicrobial, anticancer, anti-inflammation, anti-angiogenesis, anti-oxidation, antidiabetic, antihypertensive, hepatoprotective, and neuroprotective properties and immunomodulatory effects in both *in vitro* and *in vivo* experimental trials. In addition, it is an inhibitor of various enzymes, including choline acetyltransferase, acetylcholinesterase, and monoamine oxidase. Pharmacokinetic studies have demonstrated the low bioavailability, rapid absorption, and extensive metabolism of scopoletin. These properties may be associated with its poor solubility in aqueous media. In addition, toxicity research indicates the non-toxicity of scopoletin to most cell types tested to date, suggesting that scopoletin will neither induce treatment-associated mortality nor abnormal performance with the test dose. Considering its favorable pharmacological activities, scopoletin has the potential to act as a drug candidate in the treatment of cancer, liver disease, diabetes, neurodegenerative disease, and mental disorders. In view of its merits and limitations, scopoletin is a suitable lead compound for the development of new, efficient, and low-toxicity derivatives. Additional studies are needed to explore its molecular mechanisms and targets, verify its toxicity, and promote its oral bioavailability.

## 1 Introduction

**FIGURE 1:**
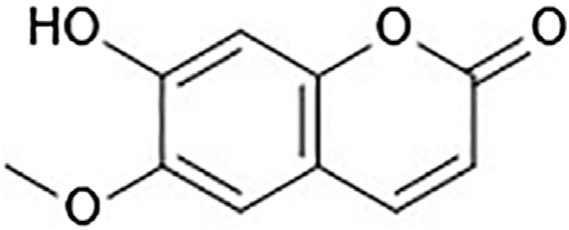
Structure of scopoletin.

In recent years, functional components of food sources have aroused considerable interest because of their benefits in preventing illnesses and promoting health (Iahtisham Ul et al., 2019). Scopoletin (6-methoxy-7-hydroxycoumarin, Figure 1) is a phenolic coumarin that is extracted from numerous medicinal and edible plants and has various pharmacological activities. Scopoletin is synthesized by diverse medicinal plants, such as *Erycibe obtusifolia* (Pan et al., 2011a), *Aster tataricus* (Ng et al., 2003), *Foeniculum vulgare* (Kwon et al., 2002), *Artemisia annua* (Tzeng et al., 2007), *Sinomenium acutum* (Shaw et al., 2003), *Melia azedarach* (Carpinella et al., 2005), and *Artemisia iwayomogi*, as well as certain edible plants, such as *Lycium barbarum* and *Morinda citrifolia* (Dou et al., 2013; Lee et al., 2014; Forino et al., 2016; Mohamad Shalan et al., 2016). In addition, it is a component of numerous fruit and vegetable crop plants, including *Avena sativa*, *Allium ampeloprasum*, *Apium graveolens*, *Capsicum annuum*, *Capsicum frutescens*, *Daucus carota*, *Cichorium intybus*, *Citrus limon*, and *Citrus aurantium*, demonstrating its low toxicity as well as its safe application as a synergistic compound together with synthetic or other natural substances, such as vanillin (Carpinella et al., 2005).

Scopoletin has attracted the attention of medicinal chemists and health professionals because of its broad range of beneficial properties, such as antibacterial, antifungal, antiparasitic, anticancer, anti-inflammation, hepatoprotective, antihyperlipidemic, antidiabetic, neuroprotective, antioxidant, anti-angiogenesis, anti-hypertensive, analgesic, anxiolytic, immunomodulatory, anti-osteoporosis, anti-allergic, anti-aging, and anti-gout activities. In addition, scopoletin is an inhibitor of various enzymes, including choline acetyltransferase, acetylcholinesterase inhibitor (Rollinger et al., 2004; Hornick et al., 2011), aldose reductase (Lee et al., 2010), γ-aminotransferase (half-maximal inhibitory concentration [IC_50_] = 10.57 μM) (Mishra et al., 2010), monoamine oxidase (Yun et al., 2001), quinone oxidoreductase (Khunluck et al., 2019), and inducible nitric oxide synthase (iNOS) (Kim et al., 1999).

**FIGURE 2:**
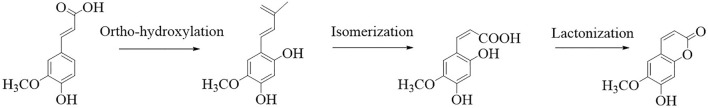
Synthesis of scopoletin (Kai et al., 2008).

Scopoletin is a hydroxycoumarin with a molecular weight of 192.7 g/mol and a melting point of 204°C–206°C. The empirical formula of the compound is C_10_H_8_O_4_. It is slightly soluble in water or cold ethanol, soluble in hot ethanol or hot acetic acid, easily soluble in chloroform, and almost insoluble in benzene. Scopoletin is biosynthesized by ortho-hydroxylation of feruloyl-CoA in *Arabidopsis thaliana* (Figure 2) (Kai et al., 2008). Scopoletin is widely distributed in medicinal plants of various families and genera, including *Malva* (Lagunas-Herrera et al., 2019; Medrano-Jiménez et al., 2019), *Cynodon* (Patel et al., 2013), *Convolvulus* (Kaur et al., 2016; Garg et al., 2018), *Artemisia* (Kang et al., 1999; Choi et al., 2012; Kim et al., 2018), *Erycibe* (Pan et al., 2010), *Canarium* (Mogana et al., 2013), and *Brunfelsia* (Oliveira et al., 2001). It is also present in the whole plant of *Viola mandshurica* W. Becker (Park et al., 2017), *Polygala sabulosa* A.W. Bennett (Meotti et al., 2006; Ribas et al., 2008; Capra et al., 2010; Pereira Dos Santos Nascimento et al., 2016), *Hedyotis diffusa* Willd. (Chen et al., 2018), and *Artemisia annua* L. (Seo et al., 2016); in the aerial parts of *Artemisia capillaris* Thunb. (Navarro-García et al., 2011), *Mitracarpus frigidus* Willd. (Lemos et al., 2020), *Solanum lyratum* Thunb. (Kang et al., 1998), and *Cirsium setidens* (Dunn) Nakai (Ahn et al., 2014); in the roots of *Hibiscus syriacus* L. (Yun et al., 2001), *Urtica dioica* L. (Nahata and Dixit, 2012), *Biebersteinia multifida* DC. (Monsef-Esfahani et al., 2013), *Gelsemium sempervirens* L. (Khuda-Bukhsh et al., 2010), *Saposhnikovia divaricata* Turcz. Schischk (Kamino et al., 2016), *Hypochaeris radicata* L. (Jamuna et al., 2015), *Argyreia specios*a L.f. (Kashyap et al., 2020), and *Angelica pubescens* Maxim. (Yang et al., 2009); the fruit of *Tetrapleura tetraptera* (Schumach. and Thonn.) Taub (Ojewole and Adesina, 1983); the flowers of *Tilia cordata* Mill. (Barreiro Arcos et al., 2006), the heartwood of *Acer nikoense* Miq. (Iizuka et al., 2007); and the inner shell of the nut of *Castanea crenata* (Noh et al., 2010). In addition, scopoletin has been analyzed in *Morinda citrifolia* L. (Kishore Kumar et al., 2017; Wigati et al., 2017; Ahmadi et al., 2019), *Fraxinus rhynchophylla* Hance (Kim et al., 1999), *Torilis radiata* (Ezzat et al., 2012), *Brunfelsia hopeana* Benth. (Oliveira et al., 2001), and *Canarium patentinervium* Miq. (Mogana et al., 2013). Table 1 summarizes the main plant sources of scopoletin and the associated bioactivities.

**TABLE 1:** Main plant sources of scopoletin and their bioactivities.

Family	Species	Parts of plant	Content W/W (%)	Bioactivity	Reference
Malvaceae	*Malva parviflora* L.	Whole plant	Not measured	Anti-hypertension and anti-AD	Lagunas-Herrera et al. (2019), Medrano-Jiménez et al. (2019)
Poaceae	*Cynodon dactylon* Linn.	Whole plant	Not measured	Anti-asthma	Patel et al. (2013)
Convolvulaceae	*Convolvulus pluricaulis* Chois.	Whole plant	Not measured	Anti-oxidant and anti-hyperlipidemia	Kaur et al. (2016), Garg et al. (2018)
Violaceae	*Viola mandshurica* W.Becker	Whole plant	Not measured	Anti-atherosclerosis	Park et al. (2017)
Rubiaceae	*Hedyotis diffusa* Willd.	Whole plant	0.0528		Chen et al. (2018)
Polygalaceae	*Polygala sabulosa* A.W. Bennett	Whole plant	0.0287, 0.112	Anti-pleurisy, anti-depression, and antinociceptive	Meotti et al. (2006); Ribas et al. (2008); Capra et al. (2010); Pereira Dos Santos Nascimento et al. (2016)
Compositae	*Artemisia annua* Linn.	Whole plant	Not measured	Anti-cancer	Seo et al. (2016)
Apiaceae	*Torilis radiata* Moench.	Aerial parts	0.0045	Hepatoprotective	Ezzat et al. (2012)
Compositae	*Artemisia capillaris* Thunb.	Aerial parts	Not measured	Downregulated MMP-1 and suppressed primary splenocyte proliferation	Kim et al. (2018); Lee et al. (2017)
Rubiaceae	*Mitracarpus frigidus* Willd.	Aerial parts	23.07 (scopoletin/extract)	Antifungal	Lemos et al. (2020)
Polemoniaceae	*Loeselia mexicana* Lamb.	Aerial parts	0.005	Antifungal	Navarro-García et al. (2011)
Solanaceae	*Solanum lyratum* Thunb.	Aerial parts	0.00023	Hepatoprotective activity	(Kang et al., 1998)
Compositae	*Cirsium setidens* Nakai.	Aerial parts	Not measured	Increased melanin synthesis	Ahn et al. (2014)
Compositae	*Artemisia feddei* Lev.	Aerial parts	0.024	Inhibited NO	Kang et al. (1999)
Asteraceae	*Artemisia iwayomogi* Kitam.	Leaves and stems	Not measured	Anti-inflammatory	Choi et al. (2012)
Convolvulaceae	*Erycibe obtusifolia* Benth.	Stems	0.022	Anti-angiogenesis and anti-arthritic	Pan et al. (2010)
Solanaceae	*Nicotiana glauca* Graham.	Leaves	Not measured	Anti-tumor	Tabana et al. (2016)
Rutaceae	*Melicope lunuankenda*	Leaves	Not measured	Inhibited α-glucosidase activity	Al-Zuaidy et al. (2016)
Rutaceae	*Aegle marmelos* Linn.	Leaves	Not measured	Decreased serum glucose	Panda and Kar (2006)
Solanaceae	*Nicotiana glauca* Graham.	Leaves	Not measured	Anti-tumor	Tabana et al. (2016)
Burseraceae Kunth	*Canarium patentinervium* Miq.	Leaves and barks	0.98	Anti-inflammatory, anti-cholinesterase, and anti-oxidant	Mogana et al. (2013)
Solanaceae	*Nicotiana glauca* Graham.	Leaves	Not measured	Anti-tumor	Tabana et al. (2016)
Convolvulaceae	*Erycibe schmidtii* Craib.	Twigs and leaves	0.066	Anti-rheumatoid arthritis	Ren et al. (2020)
Rubiaceae	*Morinda citrifolia* L.	Fruits and leaves	0.46 ± 0.05 (leaf)	Antioxidant, anti-inflammatory, anti-leukemia, anti-hypertension, anti-PD, antibacterial, anti-angiogenic, anti-gastric ulcer, gastrokinetic activity, and anti-dyslipidemic	Mandukhail et al. (2010); Mahattanadul et al. (2011); Beh et al. (2012); Nima et al. (2012); Mohamad Shalan et al. (2016); Kishore Kumar et al. (2017); Wigati et al. (2017); Ahmadi et al. (2019); De La Cruz-Sánchez et al. (2019); Narasimhan et al. (2019)
Rubiaceae	*Morinda citrifolia* L.	Fruits	0.06	Parkinson’s disease	Kishore Kumar et al. (2017)
Mimosaceae	*Tetrapleura tetraptera* (Taub)	Fruits	Not measured	Spasmolytic activity	Ojewole and Adesina (1983)
Malvaceae	*Hibiscus syriacus* Linn.	Root bark	0.0021	Anti-oxidative	Yun et al. (2001)
Urticaceae	*Stinging nettle* (*Urtica dioica* L.)	Roots	Not measured	Benign prostatic hyperplasia	Nahata and Dixit (2012)
Solanaceae	*Brunfelsia hopeana* Benth.	Roots	0.01125	Anti-hypertension	Oliveira et al. (2001)
Geraniaceae	*Biebersteinia multifida* DC.	Roots	Not measured	Anxiolytic effects	Monsef-Esfahani et al. (2013)
Loganiaceae	*Gelsemium sempervirens* L.	Roots and rhizomes	Not measured	Anticancer	Khuda-Bukhsh et al. (2010)
Umbelliferae	*Saposhnikovia divaricata* Turcz.	Roots and rhizomes	0.0039	Inhibited NO	Kamino et al. (2016)
Asteraceae	*Hypochaeris radicata* Var.	Roots	Not measured	Anti-inflammatory	Jamuna et al. (2015)
Convolvulaceae	*Argyreia speciosa* Linn.	Roots	0.56% (scopoletin/extract)	Anti-amyloidogenic	Kashyap et al. (2020)
Umbelliferae	*Angelicae Pubescentis* Maxim.	Root	0.0001		Yang et al. (2009)
Fagaceae	*Castanea crenata*	Inner shells	Not measured	Anti-oxidant	Noh et al. (2010)
Aceraceae	*Acer nikoense* Miq.	Heartwood	Not measured	Vasorelaxant	Iizuka et al. (2007)
Tiliaceae	*Tilia cordata* Mill.	Flowers	Not measured	Anti-tumor	Barreiro Arcos et al. (2006)
Oleaceae	*Fraxinus rhynchophylla* Hance.	Barks	0.0025	Inhibited NO	Kim et al. (1999)

The present review concentrates on recent research progress associated with the role of scopoletin in the prevention and/or treatment of illnesses and disorders, stressing the mechanism of its action and discussion of its toxicity and pharmacokinetic characteristics. Accordingly, recent literature concerning the bioactivity and uses of scopoletin as a chemotherapeutic agent was collated from multiple databases.

## 2 Pharmacological activities of scopoletin

**FIGURE 3:**
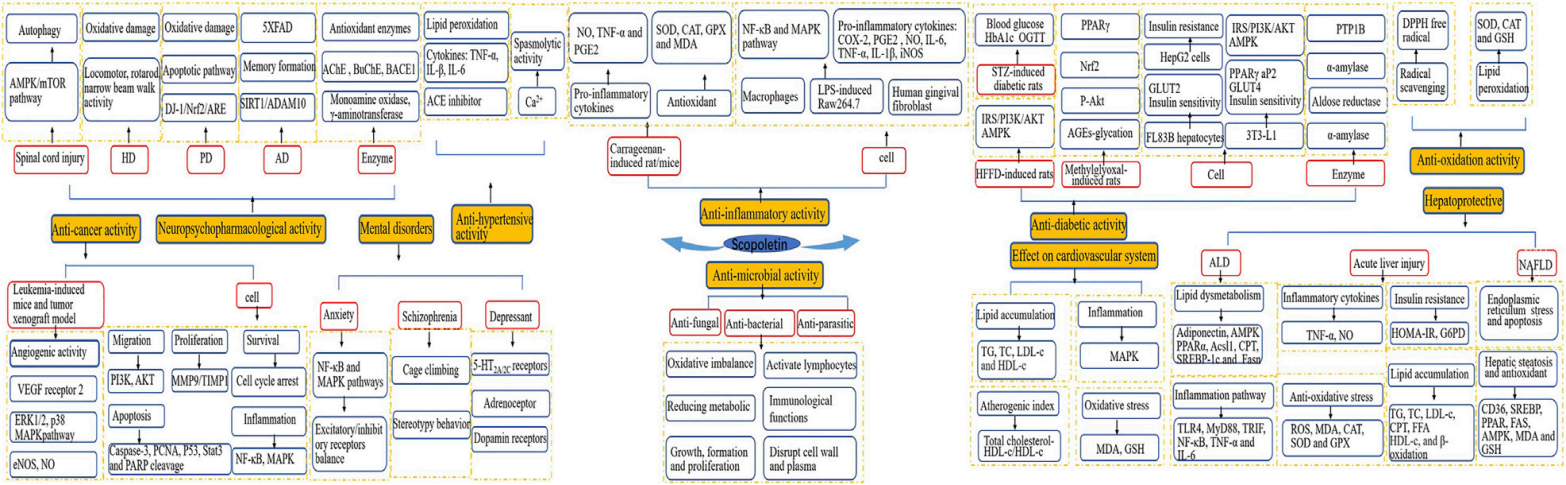
Main biological activities and possible molecular mechanisms of scopoletin.

A considerable body of evidence has proven the benefits of scopoletin in human health (Figure 3). Table 2 summarizes the health-promoting activities of scopoletin and the underlying mechanism of action for various illnesses and disorders. Details concerning the activity of scopoletin against various illnesses and disorders are discussed in the following sections.

**TABLE 2:** Treatment perspectives of scopoletin.

Disorder	Mechanism	Reference
Antifungal	Cell wall and membrane damage ↑, apoptosis ↑, oxidative imbalance ↑, and metabolic activities ↓	Das et al. (2020a); Lemos et al. (2020)
Antibacterial	Bacterial cell division ↓	Duggirala et al. (2014)
Cancer	Cell proliferative ↓	Barreiro Arcos et al. (2006); Manuele et al. (2006)
	Cell cycle arrest ↑, apoptosis ↑, and caspase-3 ↑	Liu et al. (2001)
	Caspase-3 ↑ and NF-κB ↑	Kim et al. (2005)
	Caspase-3 ↑, caspase-8 ↑, bone marrow myeloblast levels ↓, CSF3 ↓, SOCS1 ↓, PTEN ↓, TRP53 ↓, VEGF↓, IL-10 ↑, and IL-4 ↑	Ahmadi et al. (2019)
	Cyclin-D1↓, PCNA ↓, STAT-3 ↓, P53 ↑, and caspase-3 ↑	Khuda-Bukhsh et al. (2010); Bhattacharyya et al. (2011)
	Cell cycle arrest ↑ and apoptosis ↑	Asgar et al. (2015)
	NQO1↓ and cell migration ↓	Khunluck et al. (2019)
	Cell cycle arrest ↑, cleaved-3/8/9 ↑, PARP ↑, cell invasion ↓, p-PI3K ↓, and p-AKT ↓	Tian et al. (2019)
	Tumor growth ↓, vascularization ↓, and anti-vascular effect ↑	Tabana et al. (2016)
	NF-κB ↓	Seo et al. (2016)
Arthritis	Synovial angiogenesis ↓ and apoptosis ↑	Ying et al. (2009); Pan et al. (2010); Pan et al. (2011a)
	IL-6 ↓, cell proliferation ↓, MAPKs ↓, PKC ↓, and CREB ↓	Dou et al. (2013)
	COX-2 ↓	Chen et al. (2021)
	Cell proliferation ↓	Ren et al. (2020)
	IL-β ↓, TNF-α ↓, H_2_S ↓, chemoattractant protein 1↓, IL-33 ↓, preprotachykinin A ↓, and NF-κB ↓	Leema and Tamizhselvi (2018)
Pleurisy	NF-κB ↓ and MAPKs ↓	Pereira Dos Santos Nascimento et al. (2016)
Asthma	K^+^ ↓ and Ca^2+^ ↓	Patel et al. (2013)
Autoimmune encephalomyelitis	NF-κB ↓, demyelination ↓, MHC class II ↓, CD80 ↓, and CD86↓	Zhang et al. (2019)
Acute liver injury	ALT ↑, AST ↑, LDH ↑, TNF-α ↓, NO ↓, NAG ↓, MPO ↓, MDA ↓, CAT ↑, and GSH ↑	Ezzat et al. (2012)
	GSH ↑, SOD ↑, and MDA ↓	Kang et al. (1998)
	ROS ↓, CAT ↑, SOD ↑, GPx ↑, and MDA ↓	Noh et al. (2010)
Alcoholic liver	AMPK ↑, ACC ↓, SREBP-1c ↓, FAS ↓, PAP ↓, G6PD ↓, CYP2E1 ↓, SOD ↑, CAT ↑, GSH-Px ↑, GST ↑, and GSH ↑	Lee et al. (2014)
	TG ↓, adiponectin↑, AMPK ↑, SREBP-1c ↓, FAS ↓, PPARα ↑, ACSL1 ↑, CPT ↑, ACOX ↑, ACAA1A ↑, TLR4 ↓, MyD88 ↓, TRIF ↓, NF-κB ↓, TNF-α ↓, and IL-6 ↓	Lee and Lee (2015a)
	Glucose intolerance ↓, HOMA-IR ↓, serum insulin level ↓, IR ↓, PI3K ↑, GK ↑, G6Pase ↓, SOD ↑, CAT ↑, GPx ↑, H_2_O_2_ ↓, and MDA ↓	Lee and Lee (2015b)
	ALT ↓, AST ↓, TG ↓, TBARS ↓, CAT ↑, SOD ↑, GPx ↑, GR ↑, and CYP2E1 ↓	Noh et al. (2011)
Non-alcoholic fatty liver	Body weight ↓, visceral fat ↓, leptin ↓, TG ↓, TC ↓, TNF-α ↓, IL-6 ↓, IFNγ ↓, MCP-1 ↓, serum glucose ↓, insulin ↓, HOMA-IR ↓, IPGTT ↓, ERRFI1 ↑, APOA4 ↓, CYP7A1 ↓, COL1A1 ↓, MMP-13 ↓, CDKN1A ↓, and GDF-15 ↓	Ham et al. (2016)
	Body/liver weight ↓, TG ↓, TC ↓, LDL ↓, glucose ↓, ALT ↓, AST ↓, ACC ↓, SREBP-1c ↓, and p-AMPK ↑	Park et al. (2017)
Hyperlipidemia	TG ↓ and TC ↓	Kim et al. (2017a)
	TG ↓, TC ↓, and glucose ↓	Mandukhail et al. (2010)
	TG ↓, TC ↓, LDL ↓, and MDA ↓	Garg et al. (2018)
Diabetic	α-glucosidase ↓, α-amylase ↓, and postprandial hyperglycemia ↓	Abdullah et al. (2016); Jang et al. (2018)
	α-glucosidase ↓, DPPH radical activity↓, and fasting blood glucose ↓	Al-Zuaidy et al. (2016)
	Glucose uptake ↑, GLUT4 ↑, p-Akt ↑, PI3K ↑, and AMPK ↑	Jang et al. (2020)
	PPARγ 2 ↑, IR ↓, p-Akt ↑, and p-PKB ↑	Zhang et al. (2010)
	Serum glucose ↓ and G6PD ↓	Panda and Kar (2006)
	Insulin secretion ↑, blood glucose ↓, TG ↓, and TC ↓	Verma et al. (2013)
	HbA1 ↓, blood glucose ↓, FFA ↓, TG ↓, TC ↓, ALT ↓, TLR4 ↓, MyD88 ↓, TNF-α ↓, IL-6 ↓, PPARγ ↓, DGAT2 ↓, and MCP-1 ↓	Choi et al. (2017)
	AGEs ↓, TG ↓, TC ↓, LDL ↓, HDL ↑, D-lactic acid ↑, IR ↓, blood glucose ↓, Nrf2 ↑, PPARγ ↑, p-AKT ↑, PTP1B ↓, and GLUT2 ↑	Chang et al. (2015)
	AMPK ↑, IRS1 ↑, PI3K ↑, Akt ↑, TBARS ↓, LHP ↓, PC ↓, SOD ↑, GPX ↑, CAT ↑, HOMA-IR ↓, TC ↓, TG ↓, HDL-C ↑, LDL ↓, and VLDL ↓	Kalpana et al. (2019)
	AGE formation ↓ and RLAR ↓	Lee et al. (2010)
	AR ↓, oxidative stress ↓, galactitol ↑, and GSH ↑	Kim et al. (2013)
Alzheimer’s	Aβ42 fibril ↓, AChE ↓, BuChE ↓, oxidative stress ↓, and neurotoxicity ↓	Kashyap et al. (2020)
	AChE ↑, LTP ↑, LTD ↓, and memory retention ↑	Hornick et al. (2011); Das et al. (2020a)
	Microglia phagocytic ↑, PPARγ ↑, CD36 ↑, TNF-α ↓, IL-6 ↓, and amyloid plaque ↓	Medrano-Jiménez et al. (2019)
	Apoptotic ↓, ROS ↓, SIRT1 ↑, FOXO3A ↑, ADAM10 ↑, Bcl2 ↑, CAT ↑, and SOD ↑	Gay et al. (2020)
Parkinsonism	SOD ↑, CAT ↑, GST ↑, Nrf2 ↑, GR ↑, ARE ↑, DJ-1 ↑, Keap1 ↓, and GUL3 ↑	Narasimhan et al. (2019)
	Cell death ↓, striatal neuronal loss ↑, Bax ↓, Bcl2 ↑, and cytochrome C ↓	Kishore Kumar et al. (2017)
	GSH ↑, redox balance ↑, mitochondrial function ↑, and oxidative damage ↓	Pradhan et al. (2020)
Huntington’s disease	Body weight ↑, locomotor activity ↑, grip strength ↑, gait abnormalities ↑, MDA ↓, nitrite ↓, SOD ↑, and GSH ↑	Kaur et al. (2016)
Spinal cord injury	AMPK ↑, mTOR ↑, motor neuronal ↑, apoptosis ↓, autophagy ↑, beclin-1 ↑, and LC3B-positive neuronal cells ↑	Zhou et al. (2020)
Anxiety	MAOs ↓	Monsef-Esfahani et al. (2013)
	Microglia activation ↓, IL-1β ↓, IL-6 ↓, TNF-α ↓, MAPK ↓, NF-κB ↓, and GABA-T ↓	Luo et al. (2020)
Schizophrenia	Climbing behavior ↓ and stereotyped behavior ↓	Pandy and Vijeepallam (2017)
Depressant	Depression-like behavior ↓ and immobility time ↓	Capra et al. (2010)
Antioxidant	Superoxide anion ↓, SOD ↑, CAT ↑, and GSH ↑	Shaw et al. (2003)
	LDL oxidation ↓ and TBARS ↓	Thuong et al. (2005)
Anti-angiogenesis	Branching pattern of blood vessels ↓	Beh et al. (2012)
	Blood vessels ↓, tube formation ↓, proliferation ↓, and migration ↓	Pan et al. (2011b)
	VEGFR2↓, proliferation ↓, migration ↓, tube formation ↓, and ERK1/2 ↓	Pan et al. (2009)
Hypertension	Relaxed smooth muscles ↑, spasmogenic activities ↓, ACE ↓, and portal vein contractions ↓	Ojewole and Adesina (1983)
	Contractile responses ↓	Iizuka et al. (2007)
	SBP ↓, DBP ↓, TNF-α ↓, MDA ↓, IL-6 ↓, and IL-1β ↓	Lagunas-Herrera et al. (2019)
	SBP ↓ and DBP ↓	Wigati et al. (2017)
Anti-gout	Uric acid ↓	Ding et al. (2005)
Allergic	PMA ↓, IL-4 ↓, IL-5 ↓, IL-10 ↓, IFN-γ ↑, NFAT↓, GATA-3 ↓	Cheng et al. (2012)
Vitiligo	MITF ↑, tyrosinase ↑, p-CREB↑, cAMP/PKA ↑, and P38MAPK ↑	Ahn et al. (2014); Kim et al. (2017b); Heriniaina et al. (2018)
Antiaging	MMP-1↓, IL-1α ↓, TNF-α ↓, and p-P38 ↓	Kim et al. (2018)
	SA-β-Gal staining ↓, HDAC1↑, SIRT1 ↑, SIRT6↑, Nrf-2 ↑, p-FoxO1↑, and P53 ↑	Nam and Kim (2015)
Anticonvulsant	GABA transaminase ↓	Mishra et al. (2010)
Immunomodulatory	Macrophage phagocytic activity ↑	Mankar et al. (2013)
	Macrophage phagocytic activity ↑ and phagocytosis-linked genes (CDC42 ↓, FCGR1A ↓, FCGR1C ↓, ITGA9 ↓, ITGB3 ↓, PLCE1 ↓, RHOD ↓, RND3 ↓, DIRAS3 ↑, ITGA1 ↑, PIK3CA ↑, PIK3R3↑, and PLCD1↑)	Alkorashy et al. (2020)
	Splenocyte proliferation ↓ and adaptive immune cell activation ↓	Lee et al. (2017)
Osteoporosis	Osteoclastic differentiation ↓, ROS ↓, superoxide anion ↓, NF-κB ↓, and peroxyl radical-scavenging ↑	Lee et al. (2013)
Analgesic	Glutamatergic transmission ↓, TNF-α ↓, and IL-1β ↓	Ribas et al. (2008)

↓, downregulation, inactivation, and inhibition; ↑, upregulation and activation.

### 2.1 Anti-microbial activities

#### 2.1.1 Antifungal activity

The microbial population, inside and outside the human body, plays a vital role in human health because many microbes may induce illnesses. Scopoletin shows maximum antifungal activity against *Trichophyton mentagrophytes*, *Aspergillus niger*, and *Candida albicans* (Navarro-García et al., 2011). The minimum inhibitory concentration (MIC) of scopoletin against *Candida glabrata* and *Candida tropicalis* is 67.22 and 119 μg/mL, respectively, which initiates an oxidative imbalance and reduces metabolism to achieve its antibacterial effect on these two *Candida* species (Das et al., 2020). Scopoletin has antifungal properties effective against a multidrug-resistant strain of *C. tropicalis*. Its mechanism of action is interference in the synthesis of essential fungal cell components, disruption of cell walls and plasma membranes, and impairment of *C. tropicalis* biofilm growth, formation, and proliferation (Lemos et al., 2020). Recent studies have reported that scopoletin has strong antitubercular activity; the compound isolated from *Morinda citrifolia* roots exhibits high activity against *Mycobacterium tuberculosis* with an MIC of 50 μg/mL (Sam-Ang et al., 2023). The MIC of the crude ethanol extract from the stem bark of *Hymenodictyon floribundum* BL Rob. against *Mycobacterium indicum* and *Mycobacterium madagascariense* is 195 and 781.25 μg/mL, respectively (Marealle et al., 2023).

#### 2.1.2 Antibacterial activity

Several studies have reported that scopoletin is active against *Staphylococcus aureus* (Buathong et al., 2019; De La Cruz-Sánchez et al., 2019; Ramírez-Reyes et al., 2019; Chandrasekhar et al., 2023). Scopoletin exerts antimycobacterial activity against *Streptococcus pyogenes*, *Pseudomonas aeruginosa*, *P. aeruginosa* DMSC 37166, *Mycobacterium tuberculosis* H_37_Rv, *Actinomyces israelii*, *Actinomyces naeslundii*, and *Salmonella typhi* (Chiang et al., 2010; More et al., 2012; Acharya et al., 2013; Meerungrueang and Panichayupakaranant, 2014; Napiroon et al., 2018). In addition, Duggirala *et al.* reported that scopoletin inhibited both the polymerization and GTPase activity of filamentous temperature-sensitive protein Z, a target of anti-bacterial drugs, so it may be used as a lead structure for anti-filamentous temperature-sensitive protein Z drug design (Duggirala et al., 2014). Molokoane *et al.* reported that the compound from *Artemisia afra* (62.5 μg/mL) showed good activity against *Escherichia coli* (Molokoane et al., 2023).

#### 2.1.3 Antiparasitic activity

Scopoletin significantly inhibits the growth of *Plasmodium yoelii* and *Trypanosoma brucei brucei* (Mamoon Ur et al., 2014; Li et al., 2018).

#### 2.1.4 Antiviral activity

Individual fractions of scopoletin isolated from *Artemisia annua* exert strong virucidal and antiviral effects at a minimum concentration of 50 μg/mL *in vitro* and have been shown to inhibit SARS-CoV-2 infection (Baggieri et al., 2023).

### 2.2 Anticancer activity

The antitumor activity of scopoletin may result from its anti-proliferation, anti-migration, pro-apoptotic, anti-invasion, and anti-angiogenic inhibition of multiple drug resistance, regulation of the mitogen-activated protein kinase (MAPK) and PI3K/AKT/mTOR pathways, and its effect on cell cycle arrest (Antika et al., 2023).

Scopoletin shows anti-proliferative action on BW5147 murine lymphoma cells and MCF-7 human adenocarcinoma cells (Barreiro Arcos et al., 2006; Manuele et al., 2006; Nasseri et al., 2022). It exerts anticancer effects on human cervical cancer cell lines by inducing apoptosis and cell cycle arrest and inhibiting cell invasion and the phosphoinositide 3-kinase (PI3K)/protein kinase B (AKT) signaling pathway (Tian et al., 2019). It is indicated to play a role in triggering cell cycle arrest and increasing apoptosis in PC3 cells via activation of caspase-3 (Liu et al., 2001). Scopoletin has been reported to activate nuclear factor-kappa B (NF-κB), caspase-3, and PARP cleavage, leading to apoptosis of promyeloleukemic HL-60 cells (Kim et al., 2005). Scopoletin causes human melanoma cell A375 apoptosis through downregulation of cyclin-D1; proliferation of cell nuclear antigen, survivin, and Stat-3; and upregulation of p53 and caspase-3 (Khuda-Bukhsh et al., 2010). Similar outcomes have been observed for cholangiocarcinoma cells and cervical cancer cells with respect to cell cycle arrest (G_0_/G_1)_ and apoptosis induction, and an increase in cytotoxicity by co-administration of cisplatin and scopoletin is indicated (Asgar et al., 2015). Scopoletin has a significant inhibitory effect on A549 cells, with an IC_50_ of approximately 16 μg/mL (Yuan et al., 2021). In human Jurkat leukemia cells and leukemia-induced BALB/c mice, scopoletin shows anti-leukemia activity associated with cancer cell apoptosis and inhibition of inflammation and angiogenesis and mitigation of bone marrow myeloblast imbalance (Ahmadi et al., 2019). Angiogenesis plays an important role in tumor growth and metastasis (Yamakawa et al., 2018). Beh *et al.* observed that scopoletin (10, 30, and 100 nmol/egg) decreases the number of vascular branch points in a dose-dependent manner in chick embryo chorioallantoic membranes (Beh et al., 2012). Tabana *et al.* concluded that scopoletin (100 and 200 mg/kg, p.o.) shows anti-tumorigenic and anti-angiogenic activity in a nude mouse xenograft model by inhibiting vascular endothelial growth factor A (VEGFA), fibroblast growth factor 2 (FGF2), and extracellular signal-regulated kinase-1 (ERK-1) (Tabana et al., 2016). Scopoletin inhibits *in vitro* tube formation, proliferation, and migration in human umbilical vein endothelial cells and functions by obstructing VEGFR2 autophosphorylation and inhibiting ERK1/2, p38 MAPK, and Akt activation (Pan et al., 2009; Pan et al., 2011b; Cai et al., 2013). Scopoletin exposure upregulates cell cycle arrest in cancer cells, including cervical (Tian et al., 2019), cholangiocarcinoma (Prompipak et al., 2021), breast cancer (Yu et al., 2021), hepatoma, and lung cancer cells (Shi et al., 2020). A recent study has reported that matrine and scopoletin are effective ingredients of the Qinghao–Kushen combination combating liver cancer, which reduce the expression of GSK-3β in HepG2 cells and upregulate GSK-3β in HepG2.2.15 cells (Ji et al., 2022).

### 2.3 Anti-inflammatory activity

Administration of scopoletin inhibits mouse ear edema induced by ethyl phenylpropiolate, 12-*O*-tetradecanoylphorbol-13-acetate, croton oil, carrageenan, and 2,4-dinitrochlorobenzene, as well as paw and skin inflammation (Farah and Samuelsson, 1992; Muschietti et al., 2001; Ding et al., 2008; Selim and Ouf, 2012; Jamuna et al., 2015; Bak et al., 2022), which may be associated with modulation of the generation of pro-inflammatory mediators, tumor necrosis factor-α (TNF-α), interleukin-1β (IL-1β), prostaglandin E2, and IL-6, which suppresses cyclooxygenase-2 (COX-2) and iNOS expression. In lipopolysaccharide (LPS)-stimulated human gingival fibroblast and RAW 264.7 cells, scopoletin significantly inhibits the expression levels of the pro-inflammatory mediators IL-6 and TNF-α, thus prohibiting COX-2, iNOS, and nitric oxide (NO) expression (Kim et al., 1999; Kim et al., 2004; Choi et al., 2012; Kamino et al., 2016; Chaingam et al., 2021). However, the mechanism by which scopoletin influences the generation of inflammatory cytokines remains unclear. Moon *et al.* revealed that scopoletin (0.01–0.2 mM) suppresses the generation of inflammatory cytokines induced by phorbol 12-myristate 13-acetate plus A23187 by suppressing IκBα phosphorylation and degradation to obstruct NF-κB activation (Moon et al., 2007). *In vitro* assays indicate that scopoletin (100 and 200 mg/kg, i.p.) suppresses monosodium urate crystal-induced leukocyte infiltration and activation by inhibiting the synthesis and release of inflammatory mediators of activated macrophages. Scopoletin may exert anti-inflammatory effects through prevention of NF-κB signaling and the MAPK pathway (Yao et al., 2012). Pereira *et al.* reported that the anti-pleurisy effect of scopoletin is mainly mediated by inhibition of pro-inflammatory cytokines (TNF-α and IL-1β), NF-κB, and p38 MAPKs (Pereira Dos Santos Nascimento et al., 2016) and reduction in central nervous system inflammation via suppression of NF-κB signaling (Zhang et al., 2019). In addition, the regulation of the NF-κB signaling pathway reduces airway inflammation in platelet-derived growth factor BB-induced airway smooth muscle cells (Fan et al., 2022). In addition, a scopoletin-rich *Morinda citrifolia* leaf extract reduces TNF-α, IL-1β, and NO levels in serum, which relieves osteoarthritis symptoms (Wan Osman et al., 2019).

#### 2.3.1 Anti-dendritic cell activity

Rheumatoid arthritis is an autoimmune disorder characterized by synovial hyperplasia and inflammation as well as resulting in joint destruction and deformity (Sajti et al., 2004). The synovium relies on blood supply for proliferation and formation of a pannus that invades the cartilage and bone, causing osteoclast activation and cartilage and bone destruction (Kimura et al., 2007). Dendritic cells are bone marrow-derived cells that arise from lymphoid–bone marrow hematopoiesis and coordinate innate and adaptive immune responses (Collin and Ginhoux, 2019). Immature dendritic cells are preferentially localized at the lining or sub-lining layer of the rheumatoid arthritis synovium (Page et al., 2002). Scopoletin (1 and 5 μM) functionally decreases the proliferation of bone marrow immature dendritic cells (Ren et al., 2020). Scopoletin (50 and 100 mg/kg), in part, improves the clinical state of rat adjuvant-induced arthritis through ameliorating synovial inflammation and destruction of cartilage and bone, thus blocking synovial angiogenesis, triggering apoptosis of fibroblast-like synoviocytes, and inhibiting COX-2 (Ying et al., 2009; Pan et al., 2010; Chen et al., 2021; Gao et al., 2011). The anti-rheumatoid arthritis activity of scopoletin (15, 30, and 60 μM) is likely exerted by suppression of IL-6 generation by fibroblast-like synoviocytes of adjuvant arthritis and potential activation of the MAPK/protein kinase C/cAMP response element-binding protein (CREB) (Dou et al., 2013). Furthermore, scopoletin (30, 40, and 50 μM) inhibits fibroblast-like synovial cells and blocks NF-κB signal transduction, thus combating rheumatoid arthritis (Chen et al., 2022). In addition, scopoletin isolated from *Bouvardia ternifolia* (Cav.) Schltdl. inhibits NF-κB expression, thereby exerting its anti-rheumatoid arthritis action in Freund’s complete adjuvant-induced ICR mice (Zapata Lopera et al., 2022).

### 2.4 Effects of scopoletin on liver diseases

The common clinical liver diseases are mainly viral diseases caused by hepatitis B and C viral infection, drug-induced liver injury, alcoholic fatty liver disease, non-alcoholic fatty liver disease (NAFLD), cirrhosis, and liver cancer.

#### 2.4.1 Acute liver injury

Acute liver injury is the beginning of the progression of many liver diseases leading to liver failure; hence, it is a crucial research focus. Models to simulate acute liver injury mainly include a chemical liver injury model induced by carbon tetrachloride (CCl_4_), drug-induced liver injury model, drug liver injury model induced by lipopolysaccharide, and alcohol-induced alcoholic liver injury model.

Scopoletin (1, 5, and 10 mg/kg) reduces the activity of an antioxidant enzyme (superoxide dismutase, SOD) and reduced glutathione (GSH) content, inhibits the production of malondialdehyde (MDA), and resists oxidative stress during acute liver injury induced by CCl_4_ in rats so as to protect the liver (Sharma et al., 2022). Scopoletin (1, 5, and 10 mg/kg) significantly improves alanine transaminase (ALT), aspartate transaminase (AST), and alkaline phosphatase (ALP) activities in rat livers under toxicity induced by 100 mg/kg isoniazid, 300 mg/kg rifampicin, and 700 mg/kg pyrazinamide (Sharma et al., 2023).

#### 2.4.2 Chronic liver injury

##### 2.4.2.1 Alcoholic fatty liver disease

Lee et al. reported that orally administered scopoletin (0.05%, w/w) decreased lipid contents in the liver and plasma and the activities of hepatic lipogenic enzymes in alcohol plus 35% kcal high-fat diet (HFD)-induced mice. The potential mechanism for these effects was modulation of AMP-activated protein kinase (AMPK)-sterol regulatory element-binding protein 1C (SREBP-1c) pathway-mediated lipogenesis in HFD-induced mice. The hepatoprotective effect of scopoletin is associated with its stimulation of the antioxidant defense system (Lee et al., 2014). In alcohol-fed rats, scopoletin regulates AMPK and the toll-like receptor 4 (TLR4)/myeloid differentiation major response gene 88 (MyD88)/NF-κB pathway and alleviates alcoholic hepatic steatosis and inflammation (Lee and Lee, 2015). In addition, Lee et al. reported that scopoletin (0.01 and 0.05 g/L) weakened chronic alcohol-induced insulin resistance and activated the antioxidant defense system through regulation of genes involved in liver glucose and antioxidant metabolism (Lee and Lee, 2015). Scopoletin is among the predominant compounds in the inner shell of chestnut (*Castanea crenata*) and has protective effects on ethanol-induced oxidative damage *in vivo*. Its hepatoprotective effects are associated with inhibition of lipid accumulation, peroxidation, and reinforcement of the antioxidant defense system in ethanol-induced mice. Scopoletin (50 μg/mL) increases antioxidant enzyme activities (SOD, catalase, glutathione peroxidase, and glutathione reductase) in alcohol-induced HepG2 cells (Noh et al., 2011).

#### 2.4.3 Non-alcoholic fatty liver disease

In the HFD-induced obesity mice model, scopoletin (0.01% and 0.05% in diet) may mitigate NAFLD and prevent the development of liver fibrosis by regulating lipid metabolism and inflammation. The specific mechanism involves reduction of liver lipid accumulation, improvement in insulin resistance, and reduction in inflammatory factors (TNF-α, IL-6, and IFNγ), chemokine monocyte chemoattractant protein-1 (MCP-1), and leptin levels (Ham et al., 2016). Administration of scopoletin to HFD-fed mice decreases the body weight, liver weight, and serum levels for lipids and liver damage markers (ALT and AST) and regulates the AMPK/SREBP signaling pathway (Park et al., 2017). Scopoletin promotes palmitic acid-induced intracellular accumulation of triglycerides (TGs) and total cholesterol in HepG2 cells (Kim et al., 2017a). Scopoletin (6.25–50 μmol/L) inhibits endoplasmic reticulum stress and reactive oxygen species (ROS) production in primary liver cells of rats and reduces c-Jun N-terminal kinase (JNK) phosphorylation to prevent palmitic acid- and bile acid-induced liver cell death (Wu et al., 2022).

### 2.5 Effects on the cardiovascular system

#### 2.5.1 Hypotensive activity

Ojewole and Adesina, (1983) observed that scopoletin isolated from *Tetrapleura tetraptera* fruit had non-specific spasmolytic activity on smooth muscles(Ojewole and Adesina, 1983). Subsequently, Oliveira *et al.* proposed that, with regard to scopoletin derived from the roots of *Brunfelsia hopeana*, the non-specific spasmolitic action is exerted through interference with the mobilization of intracellular calcium from norepinephrine (NE)-sensitive stores (Oliveira et al., 2001), and the release of sarcoplasmic reticulum Ca^2+^ induced by NE is inhibited, resulting in vasodilation of aortic rings (Iizuka et al., 2007). Wigati et al. reported that scopoletin decreases systolic pressure (SBP), diastolic pressure (DBP), and mean arterial blood pressure (MABP) in dexamethasone-induced mice. The mechanism is associated with the activity of an angiotensin-converting enzyme (ACE) inhibitor and the antioxidant activity of scopoletin (Wigati et al., 2017). Recently, scopoletin (0.01, 0.1, 1, 2, and 5 mg/kg, p.o.) has been shown to have antihypertensive effects on chronic and acute hypertensive mice induced by administration of angiotensin II. Scopoletin decreases the pharmacodynamic parameters for SBP and DBP by 75% and 92.8%, respectively (Lagunas-Herrera et al., 2019). Among the complications associated with hypertension, the onset of intracerebral hemorrhage is a devastating stage and is the most disabling type of stroke with the highest mortality rate. Zhang *et al.* observed that scopoletin improves rat ischemia induced by collagenase injection by reducing the expression of brain edema and other inflammatory mediators, such as TNF-α and IL-1β (Zhang et al., 2021).

#### 2.5.2 Anti-atherosclerotic activity

The relevant literature clearly indicates that lipid accumulation (Cartolano et al., 2018), inflammation (Geovanini and Libby, 2018), and oxidative stress (Förstermann et al., 2017) are the most important risk factors for atherosclerosis. Scopoletin (10 μg/mL) attenuates lipid accumulation and inflammation in the aorta in HFD-induced apolipoprotein E-deficient (ApoE^−/−^) mice, which reduces vascular inflammation by AMPK activation to suppress the expression of cell-cycle regulators (cyclin and cyclin-dependent kinase adhesion molecule) in human aortic smooth muscle cells (Park et al., 2017). Subsequently, Garg *et al.* observed that scopoletin (the main component isolated from *Convolvulus pluricaulis* extract, 0.4 mg/kg) significantly decreases the levels of atherogenic lipid biomarkers, atherogenic index, and MDA and increases the levels of HDL-C and GSH in tyloxapol-induced hyperlipidemia rats (Garg et al., 2018). In addition, Batra *et al.* reported that orally administered scopoletin (1, 5, and 10 mg/kg) reduces total cholesterol, low-density lipoprotein (LDL), and TG contents and improves the plasma atherosclerosis index and Castelli risk index in the high-fructose high-fat diet (HFHFD)-induced dyslipidemia model of Wistar rats (Batra et al., 2023a).

#### 2.5.3 Anti-myocardial infarction activity

Recently, in an isoproterenol-induced myocardial infarction rat model, pretreatment with scopoletin (25 and 50 mg/kg) was observed to significantly reduce the heart-to-body weight ratio, cardiac diagnostic markers, MDA content, inflammatory markers, and apoptotic markers (Rong et al., 2023). In addition, Li *et al.* reported that scopolamine induces endothelial-dependent relaxation mediated through the NO and prostacyclin pathways, thereby alleviating acute myocardial ischemia (Li et al., 2023).

### 2.6 Antidiabetic activity

Diabetes mellitus may be the fastest-growing metabolic disease in the world. Approximately 2.5%–7% of the global population suffers from diabetes, which is a leading cause of illness and death. In diabetes, chronic hyperglycemia results from an interruption of carbohydrate and fat metabolism owing to insufficient insulin secretion, insufficient insulin function, or both (Rauf et al., 2017).

Scopoletin regulates hyperglycemia and diabetes. In the streptozotocin (STZ)-induced diabetic rat model, scopoletin has hypoglycemic and lipid-lowering effects (Verma et al., 2013; Al-Zuaidy et al., 2016). Choi *et al.* reported that scopoletin (0.01%) ameliorates hyperglycemia and hepatic steatosis in HFD- and STZ-induced diabetic mice through suppression of lipid biosynthesis and the TLR4–MyD88 pathways (Choi et al., 2017). In addition, scopoletin enhances the postprandial blood glucose levels by inhibiting the activity of carbohydrate digestive enzymes (α-glucosidase and α-amylase) in STZ-induced diabetes mice (Jang et al., 2018).

In 3T3-L1 adipocytes and high-glucose-induced HepG2 cells, scopoletin (10, 20, and 50 µM) improves insulin resistance and enhances glucose uptake by activating the PI3K/Akt signaling pathway (Zhang et al., 2010; Jang et al., 2020). Scopoletin (10 and 25 μM) improves insulin sensitivity in methylglyoxal-induced FL83B hepatocytes by activating the PPARγ/Akt pathway and restoring the plasma translocation of GLUT2 (Chang et al., 2015). In addition, improvement in insulin sensitivity in response to scopoletin (1 mg/kg/day, p.o.) can activate the AMPK and the IRS1–PI3K–Akt pathways in pancreatic β cells of high-fructose diet (HFHFD) rats and improves glucose homeostasis in HFHFD-induced diabetes rats (Kalpana et al., 2019; Batra et al., 2023b). Scopoletin (1–5 µM) stimulates insulin secretion by the KATP channel-dependent pathway in INS-1 pancreas β cells (Park et al., 2022). Furthermore, scopoletin (5, 10, 25, and 50 μM) protects INS-1 pancreatic β cells from glycotoxicity induced by high glucose and thus has potential as a drug to protect pancreatic β cells (Park and Han, 2023). Lee and Kim showed that scopoletin has potent inhibitory activity on both advanced glycation end-product (AGE) formation and rat lens aldose reductase (RLAR) in an *in vitro* bioassay, with an IC_50_ of 2.93 ± 0.06 μM and 22.51 ± 2.01 μM, respectively (Lee et al., 2010). The accumulation of AGEs is associated with an increase in the risk of fracture in patients with type 2 diabetes and has a direct adverse effect on bone quality (Yamamoto and Sugimoto, 2016). *In vitro* studies have revealed that scopoletin (1–20 µM) improves osteoclast formation in diabetes through RANKL and enhances osteoclast formation in diabetes by inducing BMP-2 and Runx2. Oral administration of 10 mg/kg scopoletin promotes the formation of bone trabeculae and collagen fibers in the femoral epiphysis and metaphysis of type 2 diabetes mice (Lee et al., 2021). Aldose reductase (AR) is a crucial rate-limiting enzyme that contributes to cataract induction among patients with diabetes. Scopoletin (10 and 50 mg/kg) mitigates diabetes cataract formation through inhibiting AR activity, polyol accumulation, and GSH generation in galactose-fed rats (Kim et al., 2013). To further explore the specific mechanism by which scopoletin alleviates diabetes retinopathy, Pan *et al* reported that scopoletin protected retinal ganglion cells from high glucose-induced damage through ROS-dependent p38 and JNK signaling cascades in a high glucose-induced retinal ganglia cell model (Pan et al., 2022). Diabetes nephropathy is among the most common microvascular complications of type 1 and type 2 diabetes; it is observed in approximately 40% of diabetes patients and is the main cause of chronic kidney disease worldwide (Vučić Lovrenčić et al., 2023). Scopoletin inhibits the proliferation of rat glomerular mesangial cells, reduces extracellular matrix proliferation and cell hypertrophy, reduces extracellular matrix protein accumulation, reduces the expression of the crucial fibrotic factor TGF-β and connective tissue growth factor, inhibits renal fibrosis, and thus improves diabetes glomerulosclerosis (Liang et al., 2021).

### 2.7 Effect of scopoletin on neurodegenerative disorders

A neurodegenerative disorder indicates the progressive loss of functions and structures and neuronal cell death arising from different conditions, such as genetic and environmental factors (Gay et al., 2020). Motor neuron degeneration is an important pathological process in many types of nervous system diseases. Motor neuron disease is characterized by chronic progressive degeneration of motor neurons. Many studies have shown that scopoletin has a neuroprotective effect, which is mainly affected via 1) inhibition of monoamine oxidase (MAO) and acetylcholinesterase (AChE), 2) reduction of oxidative damage and chronic inflammation, and 3) protection of the activity of neurotrophic factors.

#### 2.7.1 Anti-Alzheimer’s disease activity

Monoamine oxidase can be used to treat neurological disorders as a validated drug target (Carradori and Petzer, 2015). Its main function is to catalyze the oxidative deamination of neurotransmitters and biogenic amines (Edmondson et al., 2004). Yun *et al.* observed that scopoletin suppresses MAO in a dose-dependent manner with an IC_50_ value of 19.4 μg/mL (Yun et al., 2001). Furthermore, scopoletin (80 mg/kg, i.p.) is a reversible and selective MAO inhibitor causing an increase in the levels of dopamine and its metabolite (DOPAC) in the mouse brain (Mogana et al., 2013; Basu et al., 2016).

Acetylcholine (ACh) is widely distributed in the brain. The cholinergic system plays a role in crucial physiological processes, such as attention, learning, memory, stress response, wakefulness, and sleep, or sensory information (Ferreira-Vieira et al., 2016). Scopoletin can serve as an inhibitor of AChE, as indicated by the pharmacophore-based virtual screening method. The IC_50_ for AChE inhibition is 168.6 µM and 0.27 ± 0.02 mM (Rollinger et al., 2004; Mogana et al., 2013). Scopoletin shows AChE inhibitory activity in the range of 13.92%–34.18% at a concentration of 100 μg/mL (Suchaichit et al., 2018).

Neuronal cell death is an important feature of neurodegenerative disorders. In SH-SY5Y cells subject to hydrogen peroxide (H_2_O_2_) injury, scopoletin (5 μΜ) attenuates neurodegeneration via restoration of antioxidant enzyme activity, reduction in cell apoptosis, and activation of the SIRT1–ADAM10 signaling pathway, which is implicated in reduction in amyloid β (Aβ) production (Gay et al., 2020). In addition, Aβ is the main component of neuritic plaques in Alzheimer’s disease (AD) (Greenberg et al., 2020). Administration of 23 mg/kg scopoletin ameliorates the detrimental impacts of Aβ deposition on memory and learning among 5XFAD transgenic mice under a HFD diet, which is associated with microglia-enhanced phagocytic capacity and weakened microglia M1 phenotype activation (Medrano-Jiménez et al., 2019). Subsequently, Kashyap *et al.* proposed that scopoletin improves Aβ42-induced neurotoxicity and H_2_O_2_-induced cytotoxicity in PC12 cells. This effect may mediate inhibition of Aβ42 aggregation, AChE, butyrylcholinesterase, Aβ-site precursor protein-cleaving enzyme 1, MAO-B, and oxidative stress (Kashyap et al., 2020).

In conclusion, the imbalance of AChE and MAO, nerve cell death, and Aβ deposition may lead to cognitive and memory impairment. In cholinergically impaired and age-impaired mice models, scopoletin induces a significant increase in presynaptic activity-dependent acetylcholine release, enhances long-term potentiation (LTP) in the hippocampus, and exerts memory-improving properties (Hornick et al., 2011). The stimulatory role of *Convolvulus pluricaulis* extracts (500 mg/kg, scopoletin as the active ingredient), which modulate synaptic plasticity in the hippocampal cornu ammonis, enhances LTP and reduces long-term depression, which are the two major synaptic plasticity forms of memory formation (Das R. et al., 2020).

#### 2.7.2 Anti-Parkinsonism disease activity

Although the pathogenesis of Parkinson’s disease (PD) is not entirely resolved, it has been reported that excessive production of ROS, mitochondrial dysfunction, neuroinflammation, and environmental toxins can promote the loss of dopaminergic neurons in PD (Ryan et al., 2015). Rotenone-induced Sprague‒Dawley (SD) rats and SH-SY5Y cell models have shown that scopoletin inhibits cell apoptosis and oxidative stress by activating DJ-1–Nrf2–antioxidant response element (ARE) signaling (Narasimhan et al., 2019). In the same model, scopoletin attenuates rotenone-induced apoptosis of dopaminergic neurons in SD rats. The mechanism involves inhibition of the mitochondrial pathway of internal apoptosis, regulated by the Bcl2 family (Kishore Kumar et al., 2017). In addition, scopoletin (2.5 mM) is an antioxidant, reducing mitochondrial dysfunction and oxidative stress caused by an increase in ROS concentrations so as to restore motor ability and enhance the mitochondrial and cellular health of dopaminergic neurons in a *Drosophila* fly model of PD (Pradhan et al., 2020).

#### 2.7.3 Anti-Huntington’s activity

In a 3-nitropropionic acid-induced model of Huntington’s disease, administration of 20 mg/kg scopoletin attenuates motor deficits and oxidative damage in rats where it improves behavioral parameters (locomotor, rotarod, and narrow beam walking activity) and biochemical parameters (MDA, SOD, GSH, and nitrite) (Kaur et al., 2016).

### 2.8 Anti-mental disorder

Worldwide, the prevalence of mental illness is approximately 25%. The mental illness mentioned in this paper refers to the medical concept of mental pain defined in the DSM-5 diagnostic criteria traditionally used for research. However, due to its long-term effects, mental illness is also considered a disability (Littlewood, 2001; Sayce and Boardman, 2008).

#### 2.8.1 Anti-anxiety activity

*Biebersteinia multifida* root extract (45 mg/kg, i.p., including scopoletin) exhibits an anxiolytic effect that shows the same anti-anxiety effect as that of diazepam but lasts longer for 90 min (Monsef-Esfahani et al., 2013). In the Freund’s adjuvant-induced chronic inflammation anxiety mouse model, scopoletin (50 mg/kg, i.p.) exerts an anxiolytic effect through ameliorating anxiety-like behaviors, for which the mechanism is associated with suppression of the NF-κB/MAPK signaling pathways involving anti-inflammatory activities and regulation of the excitatory/inhibitory receptor balance (Luo et al., 2020).

#### 2.8.2 Anti-schizophrenia activity

Scopoletin at a specific dose of 0.1 mg/kg can alleviate the positive symptoms of schizophrenia. Scopoletin exerts anti-climbing and anti-stereotypy effects on apomorphine-induced cage climbing and methamphetamine-induced stereotypy behaviors, respectively, in mice (Pandy and Vijeepallam, 2017).

#### 2.8.3 Anti-depressant activity

Scopoletin (10–100 mg/kg, p.o.) shows particular antidepressant-like effects, as observed in the tail suspension test. Antidepressant effects are associated with the interaction of serotonergic (5-HT_2A/2C_ receptors), noradrenergic (α_1_-and α_2_-adrenoceptor), and dopaminergic (D_1_ and D_2_ receptors) systems (Capra et al., 2010).

### 2.9 Anti-oxidant activity

Scopoletin hinders oxidation in the ABTS, diphenyl-2-picrylhydrazyl (DPPH), FRAP, and β-carotene bleaching assays with half-maximal effective concentration (EC_50_) values of 5.62 ± 0.03 μM, 0.19 ± 0.01 mM, 0.25 ± 0.03 mM, and 0.65 ± 0.07 mM, respectively (Mogana et al., 2013). Scopoletin scavenges xanthine/xanthine oxidase-generated superoxide anions in a dose-dependent manner while xanthine oxidase activity is maintained and enhances the activity of endogenous antioxidant enzymes, such as SOD, catalase, and GSH (Shaw et al., 2003; Panda and Kar, 2006). Furthermore, scopoletin inhibits xanthine oxidase, maintains mitochondrial functioning to reduce ROS amounts, and suppresses LDL oxidation mediated by either Cu^2+^ or free radicals generated with an azo compound (Thuong et al., 2005). In addition, scopoletin shows antioxidant potential against DPPH (IC_50_ = 0.82 mg/mL) and NO (IC_50_ = 0.64 mg/mL) radicals (Sam-Ang et al., 2023).

### 2.10 Miscellaneous properties

#### 2.10.1 Anti-gout-lowering uric acid activity

Ding *et al.* showed that scopoletin (100 and 200 mg/kg, i.p.) causes a significant reduction in uric acid activity associated with potassium oxonate by decreasing the serum uric acid level and enhancing urine urate (Ding et al., 2005), although the mechanism is not clear. Scopoletin (200 mg/kg, p.o.) remarkably lowers the serum uric acid level of a yeast extract in potassium oxonate-induced mice; the therapeutic mechanisms are associated with inhibition of the activity of hepatic xanthine oxidase and promotion of uric acid excretion (Zeng et al., 2017).

#### 2.10.2 Anti-allergic activity

Scopoletin (50 μΜ) exerts anti-allergic activity mainly by inhibiting the production of cytokines (IL-4, IL-5 IL-10, and IFN-γ) and suppressing nuclear factor and GATA3 expression in activated T cells and PMA-/ionomycin-induced EL-4 T cells (Cheng et al., 2012).

#### 2.10.3 Anti-vitiligo activity

Vitiligo is a skin disease. The death or loss-of-function of skin melanocytes leads to partial discoloration of the skin (Pichler et al., 2006). Ahn *et al.* reported that scopoletin increases melanin synthesis in B16F10 cells by activating cAMP-responsive CREB phosphorylation and microphthalmia-associated transcription factor (MITF), resulting in an increase in the expression of tyrosinase (Ahn et al., 2014). In addition, scopoletin (40 μg/mL) stimulates melanin synthesis through activation of the cAMP/PKA/p38 MAPK pathway in B16 melanoma cells (Kim et al., 2017b). Furthermore, scopoletin (10, 20, and 25 μΜ) enhances melanogenesis responses in zebrafish and B16F10 cells, which is associated with increases in melanin content and expression of tyrosinase-related protein 1 and MITF (Heriniaina et al., 2018).

#### 2.10.4 Anti-aging activity

For human lung fibroblasts, scopoletin has anti-aging effects, which promotes autophagy induction via inactivation of p53 and enhance FoxO transportation, thereby inducing anti-aging-related autophagy and longevity (Nam and Kim, 2015). In HaCaT human keratinocytes with UVB, scopoletin (30, 100, and 300 µM) inhibits the expression of pro-inflammatory cytokines and matrix metallopeptidase (MMP)-1 by inhibiting the phosphorylation of p38 MAPK (Kim et al., 2018).

#### 2.10.5 Immunomodulatory activity

Alkorashy *et al.* demonstrated that scopoletin (50 μg/mL) stimulates U937-derived macrophages and significantly affects the expression of certain phagocytosis-linked genes (Alkorashy et al., 2020). Scopoletin (10 and 100 μΜ) suppresses ConA- and LPS-induced adaptive immune cell activation (Lee et al., 2017).

#### 2.10.6 Anti-nociceptive activity

Scopoletin, found in a *Polygala sabulosa* hydroalcoholic extract (0.01–10 mg/kg, i.p.), inhibits the acetic acid-induced visceral nociceptive response (Meotti et al., 2006). In addition, scopoletin (10 mg/kg, i.p.) counteracts nociception induced by glutamate in mice (Ribas et al., 2008).

#### 2.10.7 Anti-spinal cord injury activity

In a rat model of spinal cord injury, scopoletin (100 mg/kg, i.p.) improves locomotion 325 recovery and motor neuronal loss through stimulation of autophagy by triggering the AMPK/mammalian 326 target of the rapamycin (mTOR) signaling pathway (Zhou et al., 2020).

#### 2.10.8 Facilitating the digestion activity

Sun *et al.* preliminarily confirmed that scopoletin isolated from *Cynachum auriculatum* has an anti-functional dyspepsia effect (Sun et al., 2024). Scopoletin remarkably prevents acid reflux esophagitis production, with a similar efficiency to that of standard anti-secretory agents (ranitidine and lansoprazole) through its anti-inflammatory and anti-secretory attributes, such as its pro-kinetic activity, which can accelerate gastric emptying and intestinal transit (Mahattanadul et al., 2011). The potential mechanism is partially ascribed to the active component stimulating the 5-HT_4_ receptor (Nima et al., 2012).

#### 2.10.9 Inducing the expression of latent HIV

Reversing the incubation period of HIV-1 can promote the killing of infected cells, which is crucial for treatment strategies. In HIV-1 latently infected Jurkat T cell lines, scopoletin (2.0 mM) can significantly influence the incubation period of HIV-1 without cytotoxicity in a dose-dependent manner (Zhu et al., 2023).

#### 2.10.10 Inducing metabolomic profile disturbances

Yao *et al.* evaluated the metabolic effects of scopoletin in zebrafish embryos using non-targeted metabolomics methods. Exposure to scopoletin (2.1, 6.2, and 18.5 μg/mL) resulted in significant metabolic disorders, mainly involving phosphonate and phosphinate metabolism, vitamin B6 metabolism, histidine metabolism, sphingolipid metabolism, and folate biosynthesis (Yao et al., 2022).

#### 2.10.11 Ameliorating nephrotoxicity

Scopoletin (50 mg/kg/once daily, i.p.), via the Keap1–Nrf2/HO-1 and IκBα–P65–NF-κB–P38/MAPK signaling pathways, effectively improves renal function, oxidative stress biomarkers, and inflammatory mediators in vancomycin-treated rats (Khalaf et al., 2022).

## 3 Pharmacokinetics of scopoletin

Pharmacokinetics is the study of the time course of the absorption, metabolism, distribution, and excretion of a drug, compound, or novel chemical entity upon its administration to the body (Fan and de Lannoy, 2014). Pharmacokinetics research provides compound-/drug-specific data to determine doses and dosing routes for individual patients, minimize toxicity, and offer a cornerstone for illnesses (Visser, 2018).

### 3.1 Absorption, metabolism, and elimination

Absorption, metabolism, and elimination transformations of scopoletin are widely used for monitoring its possible effects on different lifestyle-related disorders.

Following intragastric administration of 50 mg/kg scopoletin in rats, Xia *et al.* used high-performance liquid chromatography (HPLC) to tentatively detect the parameters (*T*_max (min)_ = 10, *C*_max_ (g/m) = 8.2 ± 0.8, *T*_1/2 (min)_ = 14.1 ± 0.6, AUC_t (g min/mL)_ = 145.9 ± 11.8, and Ke _(min−1)_ = 0.051 ± 0.005) associated with the absorption process by rat plasma (Xia et al., 2007). Given the low sensitivity of the HPLC method, it is unsuitable to study the *in vivo* absorption characteristics of scopoletin in detail. Therefore, Liu et al. studied its pharmacokinetics by HPLC/tandem mass spectrometry (Liu et al., 2011; Zeng et al., 2015; Li et al., 2019). The average percentage of scopoletin excreted from urine, above the dose administered, was 14.93%, and the cumulative biliary excretion of scopoletin above the dose administered was 0.16% after oral administration of Glehniae Radix extract to male SD rats (Liu et al., 2011). The results revealed that less than 15% of the analytes unchanged from the extract were excreted in the urine and less than 1% of the analytes unchanged from the extract were excreted in the bile, indicating that scopoletin undergoes major metabolism in the body. A pharmacokinetic study on rats after oral administration of scopoletin (5, 10, and 20 mg/kg) revealed that the oral bioavailability following a dose of 5 mg/kg was 6.62% ± 1.72%, for a dose of 10 mg/kg, it was 5.59% ± 1.16%, and for 20 mg/kg 5.65% ± 0.75% in the rat plasma (Zeng et al., 2015). Pharmacokinetic studies of dog plasma after oral administration of scopoletin (10, 25, and 50 mg/kg) showed that the bioavailability was 7.08%, 5.87%, and 5.69%, respectively (Zhao et al., 2019), similar to the bioavailability of coumarin (3.40% ± 2.60%) (Ritschel et al., 1977). Thus, these results indicated the statistical similarity of the oral bioavailability among the three p.o. groups and thus was found to be independent of the delivery of the administered dose. Zhang et al. applied the UHPLC-LTQ-Orbitrap-MS method to study the pharmacokinetics of scopoletin in dog plasma after intravenous (1 mg/kg) and oral administration (10, 25, and 50 mg/kg). The main relevant measurement parameters were as follows: AUC_0-t_ (ng/h/mL) = 186.54 ± 36.45, 131.83 ± 19.23, 277.78 ± 35.12, and 528.19 ± 45.78; AUC_0-∞_ (ng/h/mL) = 197.97 ± 35.21, 140.43 ± 21.10, 284.69 ± 39.87, and 546.61 ± 51.28; *T*_1/2_ (h) = 2.05 ± 0.27, 2.36 ± 0.45, 1.87 ± 0.21, and 1.65 ± 0.45; *C*_max_ (ng/mL) = 423.23 ± 39.45, 85.47 ± 15.78, 253.78 ± 45.27, and 410.79 ± 57.19; and CL/F (L/kg/h) = 5.05 ± 0.89, 71.22 ± 15.23, 87.87 ± 15.56, and 91.47 ± 17.28 (Zhao et al., 2019). Li et al. reported that in rats administered 100 mg/kg scopoletin by gavage, the relevant pharmacokinetic parameters are as follows: AUC_0-t_ (μg/L/h) = 203 ± 29.5; AUC_0-∞_ (μg/L/h) = 206 ± 29.1; *T*_1/2_ (min): 69.6 ± 5.4; *C*_max_ (μg/mL) = 72.7 ± 8.7; and CL/F (L/kg/min) = 418.9 ± 36.8 (Tian et al., 2023). Li et al. reported that, after the oral administration of 30 mg/kg scopoline, which is a metabolite of scopoletin, significant differences in certain parameters were observed between male and female rats (*p* < 0.05), i.e., AUC (9783.33 ± 157.61 ng/mL/min vs. 12,966.66 ± 1771.97 ng/mL/min), *T*_max_ (14.00 ± 5.48 min vs. 6.67 ± 2.58 min), and CL/F (3.07 ± 0.05 L/min/kg vs. 2.36 ± 0.36 L/min/kg). Further investigation is needed to elucidate the potential mechanism of gender differences; however, the maximal excretion rates of scopoletin were 31.68 μg/h and 25.58 μg/h in male and female rats, respectively (Li et al., 2019).

Coumarin is quickly absorbed from the human gastrointestinal tract and is thoroughly metabolized by the liver, and only 2%–6% of the coumarin enters the systemic circulation intact (Ritschel et al., 1977; Ritschel et al., 1979). Scopoletin is a coumarin analog, and its rapid absorption, metabolism, and excretion from the human body may explain the poor bioavailability (Zeng et al., 2015). One study has shown that scopoletin is eliminated by first-order kinetics after intraperitoneal injection of Ding Gong Teng in mice. It showed the characteristics of a two-compartment open model: rapid absorption, rapid distribution, rapid action, and slow elimination. After intramuscular injection of Ding Gong Teng (scopoletin content: 2030 mg/L) in rabbits, the absorption showed double peaks: the first peak appeared at 8.08 min, and the concentration of scopoletin was 145.45 μg/L; the second peak appeared at 2.45 h, and the scopoletin concentration was 48.66 μg/L (Min et al., 2000). The pharmacokinetic study of Ding Gong Teng injection in rabbits showed that scopoletin was eliminated quickly, the elimination rate constant was 0.56 h, the half-life was 1.81 h, and the concentration at 4 h after administration was 5.32 μg/L. An additional study reported that scopoletin was well-absorbed in a human colon adenocarcinoma cell line model, indicating that it is well-absorbed in the gut lumen (Galkin et al., 2009). The aforementioned results suggest that scopoletin undergoes extensive metabolism in the body. Wang *et al.* determined that hepatic injury does not significantly influence the pharmacokinetics of scopoletin (Wang et al., 2018). The reason for this may be that cytochrome P450 enzymes had underwent partial change in the process of liver injury. It is also possible that scopoletin is not a P-glycoprotein substrate (Nabekura et al., 2015; Yang et al., 2015), which would explain the decrease in the bioavailability of scopoletin. The bioavailability of scopoletin is low (approximately 6.0%) (Zeng et al., 2017), which may be associated with its low water solubility and instability in physiological media. It may also reflect limited solubility, poor absorption and metabolism, or decomposition in the gastrointestinal tract (Issell et al., 2008).

Nevertheless, after oral administration of a *Hedyotis diffusa* extract (4.837 g/kg equivalent to 30.45 mg/kg of scopoletin), scopoletin was rapidly absorbed into the circulatory system in rats, and the half-life and average retention time were more than 10 h (Chen et al., 2018), indicating that the clearance rate of scopoletin in the plasma was slow.

### 3.2 Distribution

Following oral administration of 6 g/kg of Angelicae Pubescentis Radix extract to rats at the lower limit of quantification levels (2.16 ng/mL), scopoletin could not be determined in the rat plasma. Analysis of its tissue distribution showed that scopoletin was extensively distributed in multiple tissues, particularly the heart, liver, and kidneys, reflecting its pharmacological roles (Chang et al., 2013).

The pharmacokinetic deficiencies and outlook for scopoletin can be briefly summarized as follows: 1) the optimal method to investigate the pharmacokinetics of scopoletin requires clarification; 2) there are distinct differences in pharmacokinetic parameters between mice of different genders, and additional studies should be conducted to explore the underlying mechanism of gender differences; 3) the pharmacokinetic parameters of scopoletin have only been studied in mice/rat and rabbit models.

## 4 Toxicology of scopoletin

No strict boundary is proposed to portray or differentiate favorable or detrimental chemicals. The degree of harmfulness and safety seems to depend on the chemical dose. Therefore, this concept has become the hub of modern toxicology, meaning that dose determines toxicity (Hayes and Dixon, 2017).

### 4.1 Toxicity

As indicated by the acute toxicity test, scopoletin failed to generate treatment-associated mortality and abnormal performance at the limit test dose (2000 mg/kg, p.o.) for 14 days in SD rats (Tabana et al., 2016). This research shows the safety of scopoletin at the dose level, and, therefore, the LD_50_ value of scopoletin for oral toxicity is > 2000 mg/kg. Jamuna *et al.* observed rats for 14 days after administration of oral doses of 10, 50, and 100 mg/kg scopoletin and detected no obvious acute toxicity signs, no net gain or loss of body weight, or gross behavioral variation (Jamuna et al., 2015). *In vivo* experiments have administered a dose of 50–200 mg/kg (i.p.) scopoletin to SD rats and ICR mice (Ding et al., 2005; Pan et al., 2010; Yao et al., 2012). Table 3 summarizes the reported dosages of scopoletin for different animals. Thus, previous research has defined scopoletin as a relatively safe natural product, but there is a lack of long-term toxicity studies on animals. Strict experiments *in vivo* should be conducted for improved estimation of the side effects of scopoletin to ensure its safe use.

**TABLE 3:** Dose range of scopoletin examined in animal models.

Strain	Animal model	Administration approach	Experimental duration	Tested dosage	Result	Reference
SD rats	Ether-induced arthritis model	i.p.	23 days	50 and 100 mg/kg	Suppressed new vessel formation	Pan et al. (2010)
ICR mice	Murine air pouch model	i.p.	6 h	50, 100, and 200 mg/kg	Inhibited monosodium urate crystal-induced inflammation	Yao et al. (2012)
SD rats	Drug-induced liver injury model	p.o.	21 days	1.5 and 10 mg/kg	Decreased ALT, AST, and ALP levels	Sharma et al. (2023)
Wistar male rats	Type IV collagenase I was injected into the left striatum		6 weeks	100 mg/kg	Reduced the expression of brain edema and inhibited inflammation	Zhang et al. (2021)
Wistar rats	HFHFD-induced dyslipidemia model	p.o.	60 days	1.5 and 10 mg/kg	Reduced TG, TC, and LDL-H and improved plasma atherosclerosis index and Castelli risk index	Batra et al. (2023a)
Albino male rats	Isoproterenol-induced myocardial infarction model	p.o.	28 days	50 mg/kg	Reduced heart to body weight ratio, cardiac diagnostic markers, inflammatory markers, and apoptotic markers	Rong et al. (2023)
db/db mice		p.o.	10 weeks	10 mg/kg	Decreased weight and increased the serum RANKL/OPG ratio	Lee et al. (2021)
ICR mice	Potassium oxonate-induced hyperuricemia	i.p.	1 h	50, 100, and 200 mg/kg	Decreased hyperuricemia	Ding et al. (2005)
SD rats	Acute oral toxicity	p.o.	14 days	2000 mg/kg	Did not produce any considerable toxic behavioral effects	Tabana et al. (2016)
Athymic nude mice	HCT116 cell xenograft mouse model	p.o.	21 days	50, 100, and 200 mg/kg/day	Suppressed tumor growth by 94.7% relative to the vehicle-treated group	
Female Swiss albino mice	Acute oral toxicity	p.o.	14 days	10, 50, and 100 mg/kg	Possessed high safety profile	Jamuna et al. (2015)
Swiss albino mice	Cerulein-induced acute pancreatitis	i.p.	6 h	10 mg/kg	Improved acute pancreatitis	Leema and Tamizhselvi (2018)
Female Swiss mice	Carrageenan-induced pleurisy	i.p.	4.5 h	0.1, 1, and 5 mg/kg	Decreased neutrophil migration	Pereira Dos Santos Nascimento et al. (2016)
Female Swiss mice	Forced swimming test, tail suspension test, or open-field test	p.o.	1 h	0.1, 1, 10, and 100 mg/kg	Antidepressant-like action	Capra et al. (2010)
Swiss mice	Acetic acid-induced visceral nociceptive	i.p.	0.5 h	0.01, 0.1, 1, and 10 mg/kg	Improved visceral and inflammatory pain	Meotti et al. (2006)
Swiss albino mice		i.p.	4 weeks	80 mg/kg	Increased brain DA and decreased DOPAC	Basu et al. (2016)
Female Wistar rats	T4-induced	p.o.	1 week	0.5 and 1.0 mg/kg/day	Anti-hyperthyroid	Panda and Kar (2006)
Wistar albino rats	STZ-induced diabetic rats	p.o.	6 weeks	1 mg/kg	Decreased blood glucose level and lipid level	Verma et al. (2013)
C57BL/6N mice	STZ-induced diabetic rats		11 weeks	0.01%, w/w	Ameliorates steatosis and inflammation	Choi et al. (2017)
SD rats	HFFD-induced type 2 diabetes	p.o.	45 days	1 mg/kg/day	Improves insulin sensitivity	Kalpana et al. (2019)
SD rats	HFHFD-induced diabetes model	p.o.	74 days	1.5 and 10 mg/kg/day	Reversed insulin resistance	Batra et al. (2023b)
SD rats	Galactose-fed rats	p.o.	2 weeks	10, 50 mg/kg/day		Kim et al. (2013)
C57BL/6 mice	CFA-induced anxiety	p.o.	2 weeks	2, 10, and 50 mg/kg	Ameliorates anxiety-like behaviors	Luo et al. (2020)

### 4.2 Cytotoxicity

Scopoletin cytotoxicity has been assessed in numerous cell types in previous *in vitro* research, such as cancer cells, normal cells, immune cells, and nerve cells, illustrating that scopoletin is a relatively safe natural product. Table 4 summarizes the reported dosages of scopoletin for different cell lines.

**TABLE 4:** Cytotoxic effects of scopoletin in different cell lines.

Cell line	Species	Category	Detection method	Tested concentration or IC_50_	Result	Reference
PC3	Human	Androgen adenocarcinoma cell	MTT assay	33, 66, 133, 266, and 533 mg/mL (72 h), IC_50_ = 157 ± 25 mg/mL	Inhibited cell proliferation	Liu et al. (2001)
HL-60	Human	Leukemia cell line	MTT assay	0.025, 0.05, 0.1, 0.2, and 0.5 mg/mL (24 h) IC_50_ = 0.5 mg/mL	Induced apoptosis via activation of NF-κB and caspase-3	Kim et al. (2005)
A375	Human	Melanoma cell	MTT assay	6, 12, 24, 30, 48, and 60 μΜ (24 h)	Inhibited cell proliferation and induced apoptosis	Khuda-Bukhsh et al. (2010)
KKU-100	Human	Cholangiocarcinoma cell line	MTT assay	250, 300, 350, 400, and 500 μΜ (72 h) IC_50_ = 486.2 ± 1.5 µM	Induced cell apoptosis	Asgar et al. (2015)
KKU-M214	Human	Cholangiocarcinoma cell line	MTT assay	50, 300, 350, 400, and 500 (72 h) IC_50_ = 493.5 ± 4.7 µM	Induced cell apoptosis	
H69 cells	Human	Bile duct epithelial cell line	MTT assay	(20–72 h) IC_50_ > 500 µM	Lower cytotoxic effects	
BW 5147	Human	T-cell lymphoma cell line	Trypan blue exclusion method	10, 50, 100, and 500 μg/mL (72 h)	Inhibited cell proliferation	Barreiro Arcos et al. (2006); Manuele et al. (2006)
				EC_50_ = 251 ± 15 μg/mL		
MCF-7	Human	Human breast adenocarcinoma	MTT assay	297.17 ± 7.99 μg/mL	Inhibited cell proliferation	Nasseri et al. (2022)
KKU-100	Human	Cholangiocarcinoma cell line	Sulforhodamine B assay	0.08, 0.16, 0.33, 0.65, 1.3, and 2.6 mM (24–48 h), IC_50_ 24 h = 1.92 mM IC_50_ 48 h = 0.89 mM	Anti-migration effect	Khunluck et al. (2019)
DoTc2	Human	Cervical cancer cell lines	Cell counting assay	3.56, 6.12, 12.5, 25, 50, and 100 μM (24 h) IC_50_ = 25 μM	Triggered apoptosis and cell cycle arrest and inhibited cell invasion	Tian et al. (2019)
SiHa	Human	Cervical cancer cell lines	Cell counting assay	3.56, 6.12, 12.5, 25, 50, and 100 μM (24 h) IC_50_ = 15 μM		
HeLa	Human	Cervical cancer cell lines	Cell counting assay	3.56, 6.12.12.5, 25, 50, and 100 μM (24 h) IC_50_ = 7.5 μM		
C33A	Human	Cervical cancer cell lines	Cell counting assay	3.56, 6.12, 12.5, 25, 50, and 100 μM (24 h) IC_50_ = 25 μM		
HCvEpC	Human	Normal cell	Cell counting assay	3.56, 6.12,12.5, 25, 50, and 100 μM (24 h) IC_50_ = 90 μM		
KKU-100/KKU-213B	Human	Cholangiocarcinoma cell	MTT assay	1, 2, 3, and 4 mg/mL (24 h, 48 h, and 72 h)	Antiproliferative	Prompipak et al. (2021)
				IC50 = 1.06 ± 0.03 mg/mL, 2.14 ± 0.08 mg/mL		
RAW 264.7	Mouse	Murine macrophage cell line	MTT assay	1, 5, 10, 25, and 50 μg/mL, did not affect the cell viability		Kim et al. (2004)
RAW 264.7	Mouse	Murine macrophage cell line	MTT assay	1, 10, 25, and 50 μg/mL, did not affect the cell viability	Inhibited iNOS	Kim et al. (1999)
RAW 264.7	Mouse	Murine macrophage cell line	MTT assay	30, 100, and 300 μΜ (20 h)	Anti-inflammatory	Yao et al. (2012)
RA-FLS	Human	Fibroblast-like synoviocytes	MTT assay	10, 20, 30, 40, and 50 μΜ (24 h, 48 h)	Inhibited proliferation, migration, and invasion	Chen et al. (2022)
HASMCs	Human	Airway smooth muscle cells	CCK-8 assay		Inhibited proliferation	Fan et al. (2022)
Fibroblast-like synoviocytes	Mouse	Adjuvant arthritis rat cells	MTT assay	125, 250, 500, and 1,000 μΜ (24 h)		Ying et al. (2009)
Fibroblast-like synoviocytes	Mouse	Adjuvant arthritis rat cells	MTT assay	15, 30, and 60 mΜ (24 h) did not display remarkable cytotoxicity	Anti-rheumatoid arthritis	Dou et al. (2013)
INS-1 pancreatic β cells	Mouse	Pancreatic β cells	MTT assay	5, 10, 20, 25, 50, and 100 µM	Stimulated the secretion of insulin	Park and Han (2023); Park et al. (2022)
RGC-5 cell	Mouse	Retinal ganglion cell	MTT assay	62.5, 125, 250, and 500 mM (6 h)	Protected RGC-5 cells exposed to high-glucose environments	Pan et al. (2022)
RGMCs HBZY-1 cells	Mouse	Mesangial cell	MTT assay	0.01, 5, 0.1, and 1 μM (48 h)	Inhibited rat glomerular mesangial cells proliferation and ECM proliferation and cell hypertrophy	Liang et al. (2021)
SH-SY5Y cells	Human	Dopaminergic cell	MTT assay	1, 5, 10, and 50 μM (24 h)	Attenuated neurodegeneration	Gay et al. (2020)
PC12 cells	Mouse	Nerve cell	MTT assay	10, 20, and 40 μM	Anti-amyloidogenic and anti-cholinesterase	Kashyap et al. (2020)
EL-4 T cells	Mouse	T-lymphoma cell	Trypan blue dye exclusion	1, 10, 25, and 50 μM	Attenuates allergy	Cheng et al. (2012)
B16F10 cells	Mouse	Melanoma cells	Crystal violet assay	0.1, 1, 10, 20, and 50 μΜ (24 h)	Increases melanin synthesis	Ahn et al. (2014)
B16F10 cells	Mouse	Melanoma cells	MTT assay	0–40 μg/mL (24 h)	Stimulates melanogenesis	Kim et al. (2017a)
B16F10 cells	Mouse	Melanoma cells	MTT assay	0.5, 10, 25, 50, 100, 150, 200, and 250 µM (48 h)	Stimulates melanogenesis	Heriniaina et al. (2018)
IMR 90	Human	Lung fibroblast cells	MTT assay	1, 2, 4.8, and 16 μΜ (24 h)	Anti-aging	Nam and Kim (2015)
HaCaT keratinocytes	Human	Fibroblasts	MTT assay	0.5, 5, 15, 50, 150, 250, and 500 µM (24 h)	Downregulated MMP-1 expression	Kim et al. (2018)
U937	Human	Macrophage cell line	CytoTox96 LDH-release kit	10, 50, and 100 μg/mL	No detectable cytotoxic effects	Acharya et al. (2013)
U937	Human	Monocytic cell line	MTT assay	12.5, 25, 50, 100, 200, and 400 μg/mL	No detectable cytotoxic effects	Alkorashy et al. (2020)
Caco-2	Human	Colonic adenocarcinoma	WST-1 cytotoxicity assay	250 mM	No detectable cytotoxic effects	Galkin et al. (2009)
HUVECs	Human	Human endothelial cell	MTS assay	IC_50_ = 0.73 μM	Antitumor	Tabana et al. (2016)
3T3-L1 cells	Mouse	Embryo fibroblasts	MTT assay	5, 10, 25, 50, and 100 mM (24 h)	Did not affect the viability	Jang et al. (2018)

## 5 Conclusion and future research prospects

In summary, this review summarizes the multiple physiological effects of scopoletin, confirming its significant positive effects on different illnesses, as stated previously. Consequently, many therapeutic intervention measures should be proposed in accordance with the potential mechanisms of the active agent and its derivatives. Moreover, as a natural compound, scopoletin provides a safer alternative for pharmaceutical applications targeting hepatic, neural, and cancer illnesses. Considering the therapeutic activities and the weak oral bioavailability of scopoletin, a large number of its derivatives and pharmaceutical dosages can be designed. Shi *et al.* considered isoxazole-based hybrids of scopoletin as an efficient chemical modification that improved the anticancer activity of scopoletin(Shi et al., 2017). The 2-fluorobenzylpyridinium derivative is the most potent tested compound, with an IC_50_ value of 0.215 ± 0.015 μM, which is significantly ameliorated compared with that of scopoletin (Khunnawutmanotham et al., 2016). Multiple substituted 8,8-dimethyl-8H-pyrano [2,3-f] chromen-2-ones (chromeno-coumarin hybrids) have been synthesized based on scopoletin as vasorelaxing agents. Compared with the parent molecule scopoletin, the sensitivity of these derivatives to experimental tissues was increased by 29.40–70.89 times (Singh et al., 2020). In addition, Soluplus-based scopoletin micelles (Sco-Ms) have been produced using a simple thin-film hydration technique, and the oral bioavailability of Sco-Ms was enhanced by 438% compared with that of free scopoletin. Oral delivery of Sco-Ms showed distinctly higher hypouricemic efficiency in hyperuricemic mice compared with that of scopoletin (Zeng et al., 2020). Polymeric nanoparticle encapsulation of scopoletin induced massive apoptosis in the human melanoma cell line A375 (Bhattacharyya et al., 2011).

The poor solubility of scopoletin limits the oral absorption and bioavailability of the compound. Therefore, methods to improve the bioavailability of scopoletin, reduce its toxicity, develop a suitable method for administration, and improve its clinical efficacy should be the focus of future research. In addition, scopoletin is a constituent in many edible plants and food products and thus could be developed as a health food and functional food. Further research on human subjects should be conducted to guarantee its safety, and decomposition products in the human body should be assessed to ensure its safe application in treatments. Similarly, further efforts should be made to verify the effects of food supplements, explore their diverse effects on humans and elucidate the mechanisms of action.

## Figures and Tables

**Table:** 

**AGEs**	Advanced glycation end products
**AUC**	Area under the curve of the glucose tolerance test
**APOA4**	Apolipoprotein A-IV
**AMPK**	AMP-activated protein kinase
**Aβ**	Amyloid β
**ACC**	Acetyl-CoA carboxylase
**ALT**	Alanine transaminase
**AST**	Aspartate transaminase
**ADAM**	A disintegrin and metalloproteinases
**ACE**	Angiotensin-converting enzyme
**AGII**	Angiotensin II
**AR**	Aldose reductase
**AChE**	Acetylcholinesterase
**CYP**	Cytochrome P450 isoforms
**BuChE**	Butyrylcholinesterase
**BCL-2**	B-cell lymphoma 2
**BACE1**	Aβ-site precursor protein-cleaving enzyme 1
**CAT**	Catalase
**COX-2**	Cyclooxygenase-2
**CREB**	cAMP response element binding protein
**CSF3**	Colony-stimulating factor 3
**CDKN1A**	Cyclin-dependent kinase inhibitor 1A
**CIDEA**	Cell death-inducing DFFA-like effector A
**COL1A1**	Collagen type 1
**CPT**	Carnitine palmitoyltransferase
**CYP7A1**	Cholesterol 7 alpha-hydroxylase
**ERRFI1**	ERBB receptor feedback inhibitor 1
**DGAT2**	Diacylglycerol O-acyltransferase 2
**DPPH**	Diphenyl-2-picrylhydrazyl
**DA**	Dopamine
**DBP**	Diastolic pressure
**ERK-1**	Extracellular signal-regulated kinases-1
**EC**_**50**_	Concentration for 50% of maximal effect
**FFA**	Free fatty acid
**FOXO**	Forkhead box O transcription factor
**FAS**	Fatty acid synthase
**FGF2**	Fibroblast growth factor 2
**FoxO**	Family of mammalian forkhead box O
**FtsZ**	Filamentous temperature sensitive protein Z
**GK**	Glucokinase
**G6PD**	Glucose-6-phosphate dehydrogenase
**GLUT4**	Glucose transporter 4
**GR**	Glutathione reductase
**GSH**	Glutathione
**GABA**	Gamma-aminobutyric acid
**GPX**	Glutathione peroxidase
**GPT**	Glutamic pyruvic transaminase
**GDF-15**	Growth differentiation factor 15
**HbA1c**	Glycosylated hemoglobin
**H**_**2**_ **O**_**2**_	Hydrogen peroxide
**HUVECs**	Human umbilical vein endothelial cells
**HOMA-IR**	Homeostasis model assessment of insulin resistance
**HFFD**	High-fat high-fructose diet
**IFN-γ**	Interferon-y
**IC**_**50**_	Half maximal inhibitory concentration
**IPGTT**	Intraperitoneal glucose tolerance test
**IL**	Interleukin
**iNOS**	Nitric oxide synthase
**IRS**	Insulin receptor substrate
**LDH**	Lactic dehydrogenase
**LDL**	Low-density lipoprotein
**VLDL**	Very-low density lipoprotein
**LPS**	Lipopolysaccharide
**LHP**	Lipid hydroperoxide
**LTP**	Long-term potentiation
**LTD**	Long-term depression
**MAPK**	Mitogen-activated protein kinase
**MPO**	Myeloperoxidase
**MITF**	Microphthalmia-associated transcription factor
**MDA**	Malondialdehyde
**MABP**	Mean arterial blood pressure
**MMP**	Matrix metallopeptidase
**MCP-1**	Monocyte chemoattractant protein-1
**MAO**	Monoamine oxidase
**mTOR**	Mammalian target of rapamycin
**MAOs**	Monoamine oxidases
**MyD88**	Myeloid differentiation primary response gene 88
**MMP-13**	Matrix metallopeptidase 13
**MIC**	Minimum inhibitory concentration
**NE**	Norepinephrine
**NF-κB**	Nuclear factor-kappa B
**NAG**	N-acetyl-β-D-glucosaminidase
**Nrf2**	Nuclear factor-erythroid 2-related factor 2
**NFAT**	Nuclear factor of activated T cells
**NQO1**	Quinone oxidoreductase
**nAChR**	Nicotinic ACh receptor
**OGTT**	Oral glucose tolerance test
**PC**	Protein carbonyl
**PI3K**	Phosphoinositide 3-kinase
**PGE2**	Prostaglandin E2
**PPARγ**	Peroxisome proliferator-activated receptor γ
**PTP1B**	Protein tyrosine phosphatase 1B
**PAP**	Phosphatidate phosphohydrolase
**PMA**	Phorbol myristate acetate
**PCNA**	Proliferating cell nuclear antigen
**PKC**	Protein kinase C
**PTEN**	Phosphatase and tensin homolog
**ROS**	Reactive oxygen species
**RLAR**	Rat lens aldose reductase; SD, Sprague‒Dawley
**SREBP**	Sterol regulatory element-binding protein 1C
**STZ**	Streptozotocin
**SOD**	Superoxide dismutase
**SDH**	Sorbitol dehydrogenase
**SIRT1**	Sirtuin-1
**SBP**	Systolic pressure
**SOCS1**	Suppressor of cytokine signaling 1
**TC**	Total cholesterol
**TG**	Triglycerides
**TBARS**	Thiobarbituric acid reactive substance
**TLR4**	Toll-like receptor 4
**TNF-α**	Tumor necrosis factor α
**TRIF**	TIR-domain-containing adapter-inducing interferon-β
**TRP53**	Protein 53 tumor suppressor
**VEGF-A**	Vascular endothelial growth factor A
**WAT**	White adipose tissue
